# Successful treatment of HIV-related progressive multifocal leukoencephalopathy and immunological reconstitution inflammatory syndrome with intravenous human immunoglobulin: a case report

**DOI:** 10.1186/s12981-025-00793-x

**Published:** 2025-09-24

**Authors:** Can Li, Huan Wang, Shuiqing Liu, Xiaoxu Shen, Xinghua Jiang, Fangqin Liu, Baofang Zhang

**Affiliations:** The Affiliated Hospital of Guizhou Medical University, Guiyi Street No. 28, Guizhou, Guiyang China

**Keywords:** PML-IRIS, John cunningham vrus, Intravenous immunoglobulins

## Abstract

**Background:**

Progressive multifocal leukoencephalopathy–immune reconstitution inflammatory syndrome (PML–IRIS) is a high-mortality disease among patients with AIDS. It is caused by infection with the John Cunningham virus (JCV). Currently, there are no specific antiviral treatments targeting JCV. Thus, immune reconstitution remains the primary therapeutic approach.

**Case presentation:**

A 29-year-old male patient diagnosed with AIDS presented for medical evaluation after two months of antiretroviral therapy (ART), reporting symptoms of dizziness and headache. The detection of JC virus was confirmed in cerebrospinal fluid (CSF) through metagenomic next-generation sequencing (mNGS) analysis. Plain and enhanced cranial MRI scans revealed diffusely distributed nodular and patchy enhancement shadows within the brain parenchyma, consistent with a diagnosis of PML–IRIS. Given that glucocorticoids and PD-1 inhibitors may possess higher toxicity profiles and side effects compared to intravenous immunoglobulin (IVIG), which has been shown to restore immune function while causing fewer adverse reactions rapidly, a five-day regimen of intravenous IVIG infusion was administered in conjunction with continuous ART. Following this intervention, the patient showed significant clinical improvement, including reduced dizziness and headache, and improved neurological function.

**Conclusions:**

The administration of IVIG alone may be considered an effective immunologic reconstitution strategy in treating early stages of PML–IRIS associated with AIDS, despite the complexity of the disease. This approach could be attributed to direct anti-JCV effects, neutralization of toxins, inhibition of inflammatory cytokine release, and its relatively tolerable safety profile. This case report aims to serve as a reference for clinical practitioners regarding the use of standalone IVIG therapy for HIV-related early PML–IRIS management; however, further investigation is warranted to determine its efficacy in cases where PML–IRIS has been detected at later stages.

## Background

Progressive multifocal leukoencephalopathy-immune reconstitution inflammatory syndrome (PML-IRIS) refers to an inflammatory response that occurs following antiretroviral therapy (ART) in patients with acquired immunodeficiency syndrome (AIDS) or during the recovery of immune function in individuals with compromised immunity. This inflammatory response further exacerbates the clinical manifestations associated with progressive multifocal leukoencephalopathy. Epidemiological data indicated that the overall incidence of progressive multifocal leukoencephalopathy (PML) is approximately 0.9 cases per million people. However, this incidence rises to as high as 5% among AIDS patients who have not received ART. Among AIDS patients undergoing ART, the incidence of immune reconstitution inflammatory syndrome (IRIS) ranges from 15 to 40%. Nevertheless, comprehensive large-scale epidemiological data regarding the incidence of IRIS in patients diagnosed with PML are currently lacking [1]. Cranial magnetic resonance imaging (MRI) reveals “galaxy-like” lesions characterized by white matter changes accompanied by space-occupying effects and enhancement, which can aid in differentiating PML-IRIS from classic PML [2]. JC virus (JCV) infection was recognized as the primary causative agent for PML-IRIS. Although over 70% of healthy individuals harbor JCV and remain asymptomatic, genetic rearrangements in the viral genome can occur, resulting in a neurotropic strain that, under conditions of persistent immune suppression—such as HIV infection, malignancy, or use of immunosuppressants—subsequently causes demyelination of the central nervous system [2]. The current treatment for PML-IRIS primarily includes antiviral agents targeting JCV and immune reconstitution therapy [3, 4]. However, there are no specific anti-JCV drugs approved to inhibit JCV replication effectively. These therapies aim to combat the virus by activating the patient’s immune system. For instance, programmed death 1 (PD-1) inhibitors may be utilized. Nonetheless, individual responses to such treatments can vary significantly, resulting in some patients experiencing inadequate therapeutic effects. In patients with PML-IRIS, the excessive inflammatory response associated with IRIS exacerbates the patient’s condition, complicating treatment efforts and increasing overall suffering. Currently, management of IRIS predominantly involves anti-inflammatory agents like glucocorticoids. However, glucocorticoid use carries inherent risks—including exacerbation of existing infections—and may yield limited efficacy in cases of severe IRIS [5–7]. Furthermore, emerging technologies such as CRISPR/Cas9 gene editing and T-cell immunotherapy hold promise as potentially effective treatment strategies, but their benefits remain uncertain within the context of PML-IRIS at this time. Therefore, it is essential to investigate safer and more effective immunomodulatory approaches moving forward.

This article presents a case of a patient diagnosed with PML-IRIS at an early stage, who demonstrated significant improvement in clinical symptoms following the timely administration of intravenous immunoglobulin (IVIG) in conjunction with antiretroviral therapy ART. During follow-up, the clinical improvement corresponded with gradual absorption of brain lesions and an overall favorable prognosis. IVIG, an intravenous preparation composed of polyclonal antibodies, may exert its effects by neutralizing the JCV, suppressing cytokine storms, and modulating the immune response, while causing fewer adverse reactions compared to other treatments. This report aims to propose a potential new treatment option for clinical practice, particularly for patients presenting with early PML-IRIS. However, its efficacy in advanced cases requires further validation.

## Case presentation

**Fig. 1:**
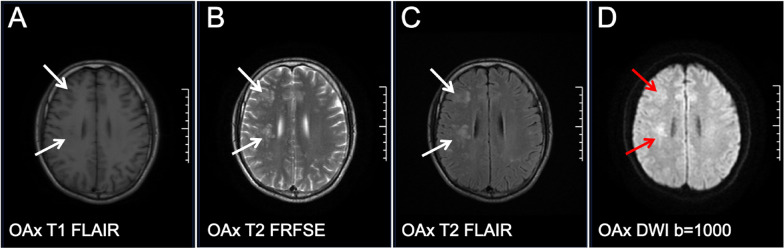
MR Plain scan results before admission (April 6, 2024). Craniocerebral MRI showed multiple nodular, lamellar and slightly longer T1 and slightly longer T2 signals in bilateral frontoparietal temporo-occipital lobe, right insula lobe, bilateral cerebellar hemispheres, and pontine, with borderless clearness, FLAIR with slightly higher signal, DWI with slightly higher signal, and ADC with slightly lower signal. A to D are MRI images at the level of OAx T1 FLAIR, OAx T2 FRFSE, OAx T2 FLAIR, and OAx DWI b=1000

**Fig. 2:**
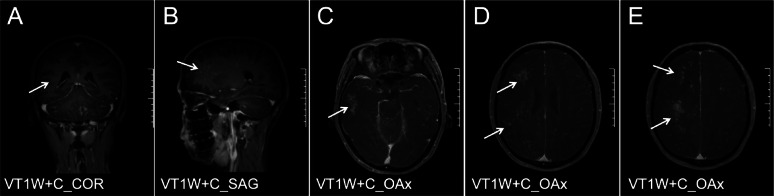
Craniocerebral MR Enhancement of patients before IVIG treatment (April 9, 2024). Small nodular and patchy enhanced shadows were diffused in the cerebral parenchyma, notably in the left cerebellum and the right frontal parietal lobe. A to E are MR Enhancement at the level of VT1W+C_COR, VT1W+C_SAG, VT1W+C_OAx, VT1W+C_OAx, VT1W+C_OAx

**Fig. 3:**
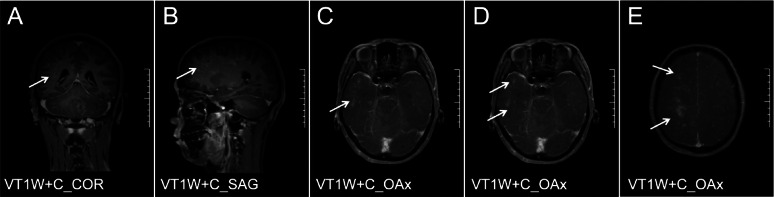
Results of craniocerebral MR Enhancement after IVIG treatment. Compared with before IVIG treatment, the absorption and enhancement of bilateral cerebellar lesions were decreased after treatment. A to E are MR Enhancement at the level of VT1W+C_COR, VT1W+C_SAG, VT1W+C_OAx, VT1W+C_OAx, VT1W+C_OAx

**Fig. 4:**
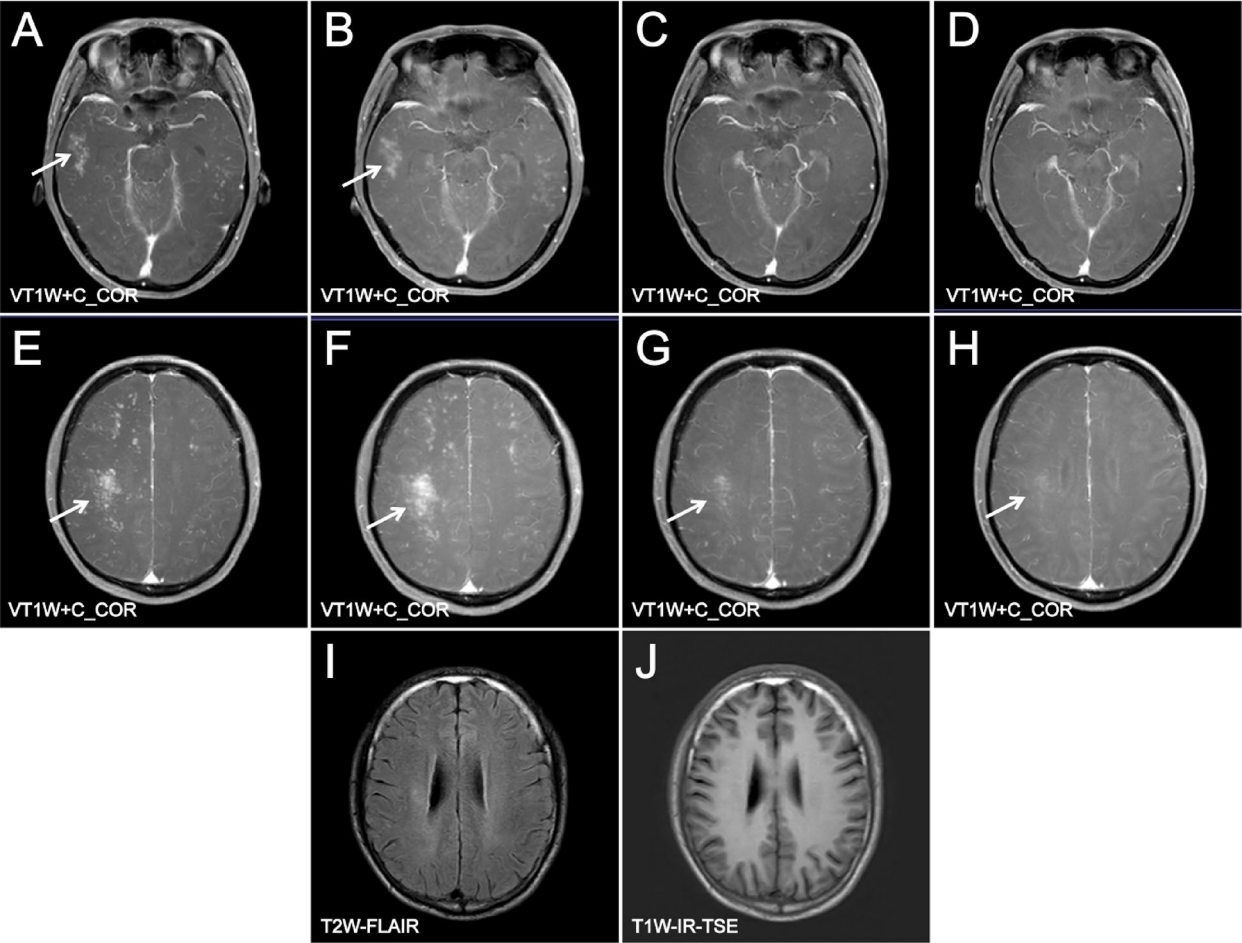
AE MR Enhancement before IVIG treatment; BF MR Enhancement after IVIG treatment; CG MR Enhancement was followed up on May 20; DH MR Enhancement was followed up on July 20. JK 12–19 Follow up with plain cranial MR scan. Compared with MR Enhancement before IVIG treatment, lesions were reduced and enhanced after 5 days of IVIG treatment. During the follow-up period, the lesions gradually absorbed, some lesions disappeared, and the enhancement continued to decrease

A 29-year-old male patient with AIDS presented with a CD4 + T cell count of 62 cells/µL and an HIV-1 RNA viral load of 14,100 copies/mL before ART. The patient was started on an ART regimen of bictegravir/emtricitabine/tenofovir alafenamide (B/F/TAF). Two months after starting ART, he experienced worsening dizziness and headaches, accompanied by an unsteady gait. Notably, the patient did not exhibit fever, convulsions, disturbances in consciousness, memory loss, or severe cognitive impairment. At this follow-up visit, upon re-examination, his HIV-1 RNA viral load was found to be < 58 copies/mL. Additionally, there was an increase in his CD4 + T cell count from 62/µL to 101/µL; the CD8 + T cell count measured at 498/µL, resulting in a CD4/CD8 ratio of 0.2 (Table 1). MRI of the brain—both plain and enhanced—revealed diffuse nodular and patchy enhancement within the brain parenchyma, particularly localized to the left cerebellum and the right frontal-parietal lobe (Figs. 1 and 2). Cerebrospinal fluid (CSF) analysis detected JCV through mNGS sequencing, with reads amounting to 20 and a relative abundance of approximately 76.92%. The diagnosis established was PML-IRIS. To mitigate immune-mediated damage while enhancing systemic immunity, as well as reducing antiviral toxicity and side effects associated with medications used alongside ART, treatment choices were made carefully. IVIG alone was administered, beginning with an initial dose of 400 mg/kg for three days, which was later adjusted downwards due to financial constraints faced by the patient. The total dose was reduced while carefully maintaining the target therapeutic level. The treatment was conducted under low-oxygen conditions to minimize stress and promote a positive response. Subsequent steps included ongoing surveillance and independent monitoring to ensure long-term efficacy. Consequently, following this intervention approach, positive clinical outcomes were displayed, reflecting significant symptom relief. Ambulation no longer necessitated assistive support during locomotion activities. Post-treatment period follow-up assessments confirmed overall improvement without any restrictions. Helper coordination in navigating ordinary living setups attained goals accordingly. The last recognized stable transition was sequentially achieved, marking a significant advancement. This concludes verifiable adjustments based on assessment baselines evaluated. The patient continues to observe progress, and further discussions are required, necessitating continual interactions and participation in decision-making and planning to guide optimized ongoing strategies. These strategies advance holistic, integrated, and individualized care trajectories. Newly developed trust-building foundations enhance overall well-being. Therapeutics were appropriately prescribed alongside guided consultations, aligning with emerging considerations and parallel strategies. These link sustainably managed conditions arising from evolving paradigms encountered in patients’ lifestyles, optimizing retention, adaptability, encouragement, and fostering resilience, which are deemed paramount. Key pivotal structures meld innovative, tailored methodologies designed inclusively, with an expected return to professional guidance. This approach explores evident maturity phases, recognizing critical intersections in provision and delivering comprehensive information regarding context-sensitive matters. It addresses discerning aspects approaching priority fronts, propelling health values integrated seamlessly and pervasively. The effort aims to break down barriers and sustain a communicable endeavor, forging accord within communities. This collective invocation of collaborative engagement synchronizes visions, nurtures uplifted horizons, and leaves a lasting imprint, transforming lives as they unfold. Re-evaluation of the enhanced cranial MRI (Fig. 3) indicated that the lesions in the bilateral cerebellar hemispheres exhibited reduced lesion size and decreased enhancement compared to previous assessments. The patient showed clinical improvement and was subsequently discharged and continued bictegravir/emtricitabine/tenofovir alafenamide (B/F/TAF) therapy after discharge. A re-examination of cerebrospinal fluid (CSF) targeted next-generation sequencing (tNGS) on May 27, 2024, revealed no detection of JCV. Follow-up evaluations were conducted on May 20, July 20, and December 19, 2024 (Fig. 4). During these follow-ups, the patient’s symptoms, including dizziness, headache, and unsteady gait, had resolved. Overall, the patient’s condition remains stable.

**Table 1:** Comparison of laboratory results before and after IVIG treatment in two patients

Variables	Pre-treatment	Post-treatment
*Blood tests*		
CD4 (cells/μL)	62	101
CD8 (cells/μL)	311	498
CD4/CD8 ratio	0.2	0.19
HIV RNA (copies/mL)	14,100	< 58
WBC (10^9^/L)	4.59	5.24
NEU% (%)	55.1	46.8
PCT (pg/mL)	<0.02	0.03
IL-6 (pg/mL)	<1.5	5.03
CRP (mg/L)	0.57	0.49
Liver and renal function	NA	NA
HBsAg, HCV-Ab, TP	NA	–
EBV DNA (copies/mL)	5.01E + 03	–
CMV DNA (copies/mL)	1.09E + 03	–
*CSF characteristics*		
Protein (mg/L)	528	518
Glucose (mg/L)	2.95	3.19
Red blood cells	0	0
White blood cells	15 × 10^6^/L	10 × 10^6^/L
*CSF microbiology*		
JCV DNA(reads)	20	Not detected

### Discussion and conclusions

Currently, there are no first-line recommended anti-JCV drugs for the treatment of PML-IRIS. The primary focus of current therapeutic strategies is on restoring the body’s immune response, which includes ART, corticosteroids, and immune checkpoint inhibitors (ICIs). Lisco et al. [3] demonstrated that JCV-specific CD4 + T cells harbor an inducible HIV reservoir. Furthermore, the expansion and recruitment of these JCV-specific CD4 + T cells to the central nervous system during PML-IRIS correlate with antigen-driven activation of memory CD4 + T cells infected with HIV, resulting in increased HIV viral load in the CSF. Thus, it is evident that continuing ART plays a crucial role in controlling HIV replication, mitigating the contribution of JCV-specific CD4 + T cells to disease progression, and facilitating immunological recovery. The efficacy of corticosteroids is controversial. As an empirical treatment, it may be beneficial in some cases of PML-IRIS [4], but it also carries potential risks such as inducing latent tuberculosis, increasing the risk of infection [5], causing osteoporosis, and metabolic disorders, and even possibly having a profound im-pact on the JCV-specific T-cell response and might compromise the control of JCV replication [8].There are also cases reporting that corticosteroids were ineffective in the treatment of PML-IRIS [9].Recently introduced ICIs have been recognized as potential agents to augment the immune response to JCV. Reports indicate [6] that patients with PML treated with pembrolizumab have achieved clinical stability, defined as no disease progression as assessed by MRI. In the study conducted by Cortese et al. [7], eight patients diagnosed with PML were treated with pembrolizumab; five of these patients demonstrated clinical improvement. Notably, JCV was cleared from the CSF of one patient [10]. Additionally, it has been reported [11] that a 64-year-old individual infected with HIV and with a history of non-small cell lung cancer—who presented with PML and received intravenous pembrolizumab treatment—experienced rapid disease progression just one week later and ultimately succumbed to the illness. To date, studies investigating ICIs have yielded heterogeneous outcomes. However, the potential risks associated with ICIs in triggering PML-IRIS as well as other related complications remain ambiguous; moreover, there is a significant gap in identifying reliable biomarkers for predicting responses to ICI therapy. Consequently, further prospective studies are warranted to ascertain whether ICIs should be incorporated into treatment regimens for AIDS patients suffering from PML.

IVIG is a biological agent derived from the plasma of healthy individuals, with IgG constituting over 90% of its content. It exhibits broad-spectrum anti-inflammatory and immunomodulatory effects. Numerous clinical studies have demonstrated that IVIG is highly effective in various neurological disorders: in Guillain-Barré syndrome (GBS) and chronic inflammatory demyelinating polyneuropathy (CIDP), IVIG functions by modulating the complement system and neutralizing autoantibodies; during myasthenia gravis crises, IVIG can rapidly neutralize acetylcholine receptor antibodies. Furthermore, IVIG is utilized to treat neuroimmune disorders such as autoimmune encephalitis and multiple sclerosis [12]. In our study, two patients were promptly diagnosed with PML-IRIS. Considering the ongoing severe inflammatory response and the potential genetic mutations of the JCV that may influence disease progression, we propose that, based on extensive experience with IVIG’s application in neuroimmune diseases and its multiple mechanisms of action, IVIG offers distinct therapeutic benefits for treating PML-IRIS, especially for patients requiring rapid inflammation control. The most common side effects—including headache, fever, limb pain, nausea, vomiting, injection site discomfort, renal impairment, elevated transaminases, and hyponatremia—tend to be mild and manageable during clinical treatment. Consequently, we administered IVIG to these two patients over five days. Following treatment initiation, symptoms were significantly alleviated, and the patient’s prognosis improved remarkably. During this period, no adverse drug reactions related to IVIG were observed. The utilization of this treatment regimen was primarily based on the hypothesis that IVIG therapy for PML-IRIS is hypothesized to exert its effects through several mechanisms: (1) Direct neutralization of JCV toxins: Research indicates that antibodies against JCV are found in the serum of approximately 50–70% of healthy individuals. As a polyclonal antibody formulation, IVIG may contain neutralizing antibodies against JCV. In vitro studies have demonstrated that IVIG can significantly inhibit JCV infection in glial cells [13]. (2) Regulation of inflammatory responses: IVIG is capable of binding to the inhibitory receptor FcγRIIB via its Fc segment, leading to downregulation of inflammatory pathways such as NF-κB and reducing the release of pro-inflammatory mediators such as TNF-α and IL-6. In cases of HIV-associated encephalopathy, administration of IVIG has been shown to decrease CSF levels of IL-6 by more than 40% [14]; (3) Promotion of immune homeostasis: IVIG contains natural anti-idiotypic antibodies that can neutralize pathological autoantibodies—a mechanism thoroughly validated in conditions such as GBS and CIDP [15]. This report presents a case of a patient with early HIV-associated PML-IRIS who achieved substantial clinical improvement through the administration of IVIG in conjunction with ART. It is important to note that, as a case report, this study has inherent limitations, including a small sample size and the absence of a control group; therefore, caution should be exercised when generalizing these results clinically. Although IVIG demonstrated favorable safety profiles during treatment—with no significant adverse reactions reported in this cohort—potential risks must still be taken into account. These may include allergic reactions (which occur in approximately 1–3% of cases), thrombotic events (particularly in patients receiving high doses or those with predisposing risk factors), and rare occurrences of aseptic meningitis [16, 17]. Our findings suggest that IVIG at a dosage of 400 mg/kg/day for 5 days may serve as an effective treatment option for individuals presenting with early PML-IRIS. However, its efficacy, optimal duration, and dosing regimen in advanced cases require validation through larger-scale controlled studies. Clinicians are advised to make individualized treatment decisions by carefully weighing the benefits against the potential risks associated with IVIG therapy while closely monitoring for any adverse reactions.

## Data Availability

No datasets were generated or analysed during the current study.
